# An adapted enhanced social prescribing intervention vs. usual care in the treatment of loneliness in cancer survivors: Study protocol for a feasibility pilot randomized controlled trial

**DOI:** 10.1371/journal.pone.0350956

**Published:** 2026-09-18

**Authors:** Brooke Ike, Allison Cole, Brennan Keiser, Alison Tu, Madison Holcroft, Sebastian Tong

**Affiliations:** Department of Family Medicine, University of Washington, Seattle, Washington, United States of America; Karolinska Institutet, SWEDEN

## Abstract

**Introduction:**

Loneliness has increased substantially in the past decade and adversely affects the health and wellbeing of cancer survivors. Cancer diagnosis and treatment can lead to profound negative changes in health status and social support. In addition to prevention and detection of recurrence and new cancers, survivorship care also requires attention to managing financial, psychological, and physical wellbeing after cancer treatment. Multiple efficacious interventions exist for loneliness, including Social Prescribing, a UK-based model that supports individuals in improving their social connections. However, none have been adapted to meet the needs and preferences of cancer survivors.

**Design:**

This feasibility study protocol is reported according to the SPIRIT 2013 and 2025 statements. We adapted an efficacious Social Prescribing intervention to target loneliness in adult cancer survivors, using the ADAPT-ITT process. This protocol describes plans to conduct a feasibility study in preparation for a randomized controlled trial of the Enhanced Social Prescribing intervention. Participants will be randomized on a 1:1 ratio to usual care (brochure) or Enhanced Social Prescribing (intervention). We will recruit 36 participants from two rural and rural-serving primary care practices in the Northwest U.S.. Eligible individuals are age 18 or older, screen positive for loneliness on the 3-item UCLA Loneliness Scale, received a diagnosis of cancer in the past seven years, and speak English. The Enhanced Social Prescribing intervention meets one hour weekly for nine weeks. The primary outcome is participant-reported loneliness as measured by the UCLA Loneliness Scale. Secondary outcomes include participant reported social connection, self-efficacy, quality of life. Self-reported receipt of guideline concordant cancer survivorship care is an exploratory outcome. We will also measure Reach, Implementation, and Acceptability/Appropriateness of the Enhanced Social Support intervention. Using an intent to treat analysis, participants will be analyzed according to their assigned group. We will use linear mixed models to estimate differences between the intervention arm and control arm, and changes in outcomes over the study period. This study protocol is registered at clinicaltrials.gov (NCT07418437).

**Anticipated results:**

We anticipate successful recruitment of 36 participants and delivery of the Enhanced Social Prescribing intervention to 18 participants.

**Discussion:**

This study will demonstrate the feasibility of testing the Enhanced Social Prescribing intervention and provide preliminary data and procedures for a larger-scale randomized controlled trial to test the effectiveness and study the implementation of Enhanced Social Prescribing.

## Introduction

Loneliness has increased substantially in the past decade [1,2] and adversely affects the health and wellbeing of cancer survivors [3,4]. Loneliness is not a unitary construct, but a multidimensional, subjective experience encompassing distress related to perceived isolation, unmet emotional needs, and a lack of meaningful connection—regardless of the presence or absence of social contact [5]. Its prevalence in the U.S. is increasing; approximately one-third of the U.S. adult population reports feelings of loneliness [6]. Given that loneliness is associated with poorer overall mental and physical health, and risk of premature mortality, effective interventions to address loneliness are needed [7,8].

Cancer diagnosis and treatment can lead to profound negative changes in health status and social support [9,10]. An estimated 36% of the more than 18 million cancer survivors in the United States experience loneliness and social isolation [3]. Research demonstrates that cancer survivors who are racial/ethnic minorities and/or live in rural and underserved communities are even more likely to be lonely, suggesting interventions tailored to these populations may be needed [3].

Cancer survivorship care is complex and includes prevention and detection of recurrence and new cancers, monitoring and amelioration of symptoms related to cancer and its treatment, and management of chronic conditions. Cancer survivorship care also should address financial, psychological, and physical wellbeing after cancer treatment [3,11,12]. In the general population, loneliness is a major risk factor for common chronic medical conditions including hypertension [13–15], cardiovascular disease [16], heart failure [17], diabetes [18,19], obesity [18–20], depression [21], and cognitive decline [22]. Acknowledging cancer diagnosis, treatment, and survivorship as a chronic condition, this study will also explore the degree to which reducing loneliness may improve well-being and uptake of guideline concordant care among cancer survivors. Cancer survivors have unique sources of loneliness that may not be shared or understood by family members, friends, or the general community. These include cancer-related fears and worries, diagnostic and treatment concerns, and lack of or insufficient social support during their cancer treatment journey [23,24]. A systematic review and meta-analysis on loneliness in adult cancer survivors published in 2021 reported limited interventional studies to date on loneliness among cancer survivors [7]. Studies done since then have mostly been small, pilot studies and not tested with rural populations [25,26].

Multiple efficacious interventions exist for loneliness. A meta-analysis of interventions found efficacy in reducing loneliness through interventions that improved social skills, enhanced social support, and increased opportunities for social contact [27]. Building from the evidence and recognizing the substantial problem of loneliness on health, the 21st U.S. Surgeon General, Dr. Vivek Murthy, published an advisory on the loneliness epidemic that highlights three vital components of social connection: structure, function and quality [1,2]. Structure refers to the type, number and variety of relationships; function refers to the ways in which relationships serve an individual’s various needs (e.g., emotional, social and/or spiritual); and quality refers to the positive and negative aspects of relationships and interactions and how it is perceived by the individual. This model is adapted from Holt-Lunstad’s framework for understanding why social relationships are important for mental and physical health [28]. Social prescribing is a United Kingdom-based model to address loneliness [29]. In social prescribing interventions, patients are referred by general practitioners and then partner with lay and/or peer workers to develop personalized strategies to improve support and connection [30–35]. While evidence supports its’ efficacy, social prescribing has not been applied widely outside the United Kingdom and Europe [34,35]. Furthermore, it has not been tested specifically for adult cancer survivors with loneliness.

The aim of this study is to conduct a feasibility pilot randomized controlled trial to test, in adult cancer survivors who receive care in rural and rural-serving primary care practices, a loneliness intervention that draws on the principles of social prescribing. We will enroll 36 adult cancer survivors who are experiencing loneliness, and who will be randomized to either Enhanced Social Prescribing (n = 18) or usual care (n = 18). We will collect outcomes on loneliness, social connection, self-efficacy, quality of life, and quality of cancer survivorship care. Our goal is to improve overall wellness and quality of life among cancer survivors and demonstrate pilot efficacy of a novel, adapted program that may improve survivorship care outcomes.

## Methods

The approved study protocol is provided in Supplement 1

### Trial status and timeline

This study protocol is registered at clinicaltrials.gov (NCT07418437). As of March 2026, we have begun enrollment of participants. Full enrollment is anticipated by 9/30/2026. All data collection is anticipated to be completed by 12/31/26. Results of primary outcomes analysis is expected by 3/31/2027. The timeline of study data collection is provided in Supplement 3.

Prior to the feasibility study, we engaged adult cancer survivors to collaboratively adapt an intervention, building on the social prescribing model and using the ADAPT-ITT framework [36]. The Enhanced Social Prescribing intervention builds on the social prescribing model and also includes psychosocial educational support and is delivered in a group setting.

### Design

This feasibility study protocol is reported according to the SPIRIT 2013 and 2025 statement [37,38]. The SPIRIT checklist is provided in Supplement 1. We used the ADAPT-ITT process to conduct an intervention adaptation phase will follow with a randomized controlled trial (RCT) to test an Enhanced Social Prescribing intervention that targets loneliness in adult cancer survivors. We engaged adult cancer survivors and topical experts to adapt the intervention for a feasibility study. ADAPT-ITT is an 8-step approach to adapting existing evidence-based interventions to novel populations. The steps include: 1) Assessment, 2) Decision, 3) Administration, 4) Production, 5) Topical Experts, 6) Integration, 7) Training and 8) Testing [36]. The methods and results of the adaptation and intervention development phase will be reported separately.

Consistent with principles of hybrid implementation designs [39], this feasibility study prioritizes implementation outcomes—feasibility, acceptability, and appropriateness—while collecting preliminary clinical outcome data. Participants will be randomized on a 1:1 ratio to usual care (brochure) or Enhanced Social Prescribing (intervention). Outcome measures will be completed at baseline, 9 weeks (immediately post-intervention), and 17 weeks following study entry.

### Setting

The WWAMI (Washington, Wyoming, Alaska, Montana and Idaho) region Practice and Research Network (WPRN) is a practice-based research network of approximately 140 primary care practices across the 5-state region that agree to be engaged in relevant research. The practices include federally qualified health centers, rural health centers, large health systems and private practices and serve a diverse group of patients. To ensure participation from rural cancer survivors, we elected to invite all rural and rural-serving primary care practices in the WPRN to consider participating. Invitations to participate were shared electronically with champions (physician representatives to the WPRN) from each eligible rural or rural-serving health system (n = 19). Champions were instructed to respond by email with interest. The study PI and research coordinator met with interested champions (4) to share more details about the study and affirm interest/eligibility. Three interested sites affirmed willingness and capacity to support recruitment and two enrolled in the study. We limited participation to two sites based on anticipated sample size and feasibility. The practices will be reimbursed for their work with recruitment, engagement, and data provision/collection.

### Sample size calculation

Because this is a feasibility pilot study, no sample size calculations were performed. 36 participants was selected as an enrollment goal based on ensuring sufficient enrollment for at least two cohorts for the enhanced social prescribing intervention and matching enrollment to the available resources and timeline. The estimates of baseline and outcome measures from this study will be used to calculate sample size for a full-scale trial.

### Characteristics of participants and eligibility criteria

This study will include 36 participants who are 18 years of age or older at the time of enrollment, screen positive for loneliness (score of ≥ 6 on the 3-item UCLA Loneliness Scale), received a diagnosis of cancer in the past seven years, speak English, and have had a primary care clinic visit at one of the partnering primary care organizations in the past two years. The 3-item UCLA Loneliness Scale is a validated screening survey that asks about feelings of lack of companionship, feelings of being left out, and feelings of isolation from others [40]. Each item is scored on a scale of 1–3 with total score ranging from 3–9. We chose the 3-item screener instead of the 20-item UCLA Loneliness Scale because it can pragmatically be used in care settings, and we have successfully used this scale for clinic-based and text-based recruitment in previous studies [41,42].

We will exclude patients who are currently living in an inpatient or rehabilitation setting, have scheduled or planned surgery or inpatient hospital admission in the next 3 months, have severe or moderate cognitive impairment or active psychosis.

### Variables to be measured

#### Demographic information.

In the baseline survey, we will administer a demographic survey that asks patients to answer questions about age, sex at birth, gender identity, race/ethnicity, health insurance status, type of cancer and age at cancer diagnosis, zip code of primary residence, sexual orientation, living situation and employment status. These will not be asked at follow-up.

#### Consent, Randomization, concealment of allocation, and blinding.

Interested potential participants will schedule a telephone call with the research coordinator to learn more about the study. After confirming study eligibility and sharing consent information, the research coordinator will obtain the participant’s verbal informed consent to participate in the study. Following verbal consent, participants will be sent an electronic survey via REDCap to review the full electronic consent information form and then subsequently complete the baseline survey. Participants will be required to review the electronic consent information form prior to initiating the baseline line survey. If participants prefer to, they may elect to complete the baseline survey over the phone with the research coordinator. Participants will be considered enrolled in the study upon completion of the baseline survey. After enrollment, a research coordinator responsible for enrollment and study operations but not involved in data collection or analysis will randomize them in a 1:1 ratio to either Enhanced Social Prescribing intervention or enhanced usual care using random permutated blocks [43]. Randomization will be conducted using REDCap. Procedures to ensure allocation concealment include using parallel structures in REDcap for each arm, which reduces the possibility that study personnel interacting with the database can infer treatment assignment.

Enrollment and study coordination staff will not be blinded, as they will be responsible for configuring the randomization scheme in REDCap and assigning participants to study arms following a review of the electronic consent information and completion of the Baseline survey. Because of the nature of the intervention and enhanced usual care, participants will not be blinded to treatment arm. To reduce risk of bias in assessing primary and secondary outcomes, the biostatistician and analyst conducting analyses will remain blinded to treatment assignment until primary outcome analysis results are complete. Outcome data will be collected using electronic surveys administered via REDCap. A team member not involved in enrollment and randomization will conduct any follow-up calls to collect outcome data from individuals without email or who do not complete the outcome electronic survey. The study biostatistician and analyst will remain blinded by being restricted from accessing the randomization dashboard and associated forms within the REDCap project. We do not anticipate any circumstances in which the biostatistician or analyst would need to be unblinded.

#### Recruitment.

We will use a multimodal recruitment strategy in partnership with the two health systems (n = 3 primary care clinics) that is well established by our research group [44,45]. One clinic is located in Washington state and one clinic is in the Northwest region, but outside of Washington state. The strategies include flyers, provider referrals, and/or electronic health record (EHR) query. We will use our patient advisory groups to help inform strategies for recruitment within these recruitment strategies.

**Provider referrals**: Prior to the beginning of the trial, the study PI will give a presentation to providers and staff at participating clinics during a regularly scheduled staff meeting (or another meeting as preferred by the clinic staff) on the study, eligibility criteria and intervention. Providers will be given flyers that include a link, QR code, and telephone number to the study interest form, to inform patients about the study. Providers will be able to hand out these flyers to patients. Interested patients will then be directed to either access the interest form electronically and complete it independently or reach out to the study coordinator by phone, email and/or text if they have any questions.

**EHR query and outreach**: We will provide parameters information to participating clinics that will conduct EHR queries to identify adult (age 18 or older) patients with a history of cancer (ICD-10 C00-D49). The research team will partner with the clinic to send information about the research study via email, mail or patient portal to eligible patients. The communication will instruct interested patients to complete the interest form electronically.

After patients complete the interest form, they will be directed to complete a brief screening questionnaire to assess eligibility. Patients will be given USD 15 for completing the screening questionnaire form. The screening questionnaire includes the 3-item UCLA Loneliness Scale and demographic questions and patient responses will determine study eligibility. Upon completion of the screening questionnaire, a research coordinator will contact the participant to schedule the initial study phone call for formal eligibility confirmation, consent, and distribution of electronic baseline enrollment survey.

We will continue the recruitment strategies above until we reach our goal of 36 participants enrolled. Participants will be allocated to one of two study arms of roughly equivalent size. Recruitment for this study opened on 15/03/2026 and will last for six months (14/09/2026) or until recruitment target (n = 36) is reached, whichever comes first.

## Outcomes

### Assessment of trial feasibility

#### Research procedures feasibility.

Research procedures feasibility outcomes include number of individuals screened, number of individuals enrolled, time to first enrollment, time to reach enrollment milestones (25% target enrollment, 50% target enrollment, 75% target enrollment, 100% target enrollment), completeness of baseline and follow-up data, rate of participant drop-out or withdrawal, and the proportions of participants randomized to enhanced social prescribing that attended each scheduled session. Progression criteria for feasibility assessment are shown in Table 1.

**Table 1:** Progression criteria for feasibility assessment.

	Green	Yellow	Red
50% Enrollment	3 months	6 months	9 + months
Baseline data collection	100%	90%	50% or less
Drop out	<1	1-2 participants	N/A*
Intervention delivery	75% of participants	60% of participants	<60% of participants

• If greater than 2 participant drop outs are noted during the course of the study, the team will undertake full review of the study protocol and procedures to identify and mitigate design features that may be contributing to drop out.

#### Reach.

We will measure reach of study enrollment and reach of intervention participation. Reach of study enrollment will be measured by calculating the proportion of patients who indicated initial interest in the study (e.g., by responding to a study flyer or invitation) that enrolled in the study. We will also report the proportion of interested participants that screened eligible and reasons for ineligibility. We will report the demographic characteristics (age, sex, race/ethnicity, rurality, type of primary cancer, years since cancer diagnosis and household income) of enrolled participants to explore if participants are representative of the adult cancer survivor population and whether specific subpopulations are more easily reached, eligible and enrolled in our study.

#### Implementation.

We will measure participants’ completion and adherence to the intervention by tracking participant attendance at sessions, successful completion of various study activities in groups, interventionist-rated participant engagement and participant reported follow-up with plans developed. These data will be derived from interventionist notes as well as questions in the follow-up survey that will only be asked of those in the intervention arm of the study. We will also measure fidelity to the Enhanced Social Prescribing intervention through interventionalist field notes and fidelity checklists. Each week, connection navigators will be asked to complete fidelity checklists based on that week’s scheduled activities in the manual and take notes on what happened as scheduled, unanticipated events and other observations.

#### Acceptability/Appropriateness.

We will also measure the acceptability and appropriateness of the intervention in post-intervention surveys with the 18 participants that complete the intervention with the validated Acceptability of Intervention Measure (AIM) and Intervention Appropriateness Measure (IAM) measures [46]. participants will not be mixed

### Patient-reported Outcomes

#### Loneliness (primary outcome).

The UCLA Loneliness Scale, Version 3, consists of 20 items that ask patients to identify “how often they feel” followed by a positive or negative description of social interactions and perceptions, with participants ranking the frequency as never, rarely, sometimes and always (each item scored from 1 to 4). The scale is scored from 20–80 with 20 being the least and 80 being the most lonely. This scale was chosen over other loneliness scales since it is recommended by the PhenX toolkit [47], has been extensively validated [48], and has been used in previous loneliness intervention studies as an outcome measure [27,49]. We have successfully used this scale in our other interventional loneliness studies with targeted populations [50,51].

The UCLA Loneliness Scale captures social and emotional dimensions, such as perceived scarcities in social networks and intimate relationships, but does not address existential aspects of loneliness [52]. Existential loneliness, characterized by a deep feeling of separateness from others and the world, is reported among people in life-threatening situations [53], and is relevant to cancer survivors who experience existential questions of meaning and identity [54]. To capture this dimension, the validated 6-item Brief Scale of Existential Loneliness (B-SEL) will be used to assess existential loneliness as a distinct but complementary construct alongside the UCLA scale [55].

#### Social connection (secondary outcome).

Social connection will be measured by the Lubben Social Network Scale – 6. It is a well-validated 6-item questionnaire that asks about interaction and trust with family and friends in the past month. The scale is scored from 0 to 30 with each question asking about number of friends or family “seen or heard from at least once a month,” “at ease with that you could talk about private matters” or “close to such that you could call on them for help” on a ranked scale of 0 (none) to 5 (9 or more) [56].

#### Self-efficacy (secondary outcome).

Self-efficacy is the belief in one’s ability to cope with a broad range of stressful and challenging demands. The Generalized Self-Efficacy Scale has 10 items with questions about ability to cope and problem solve with responses ranging from 0 (not at all true) to 4 (exactly true) [57].

#### Quality of life (secondary outcome).

The European Organisation for Research and Treatment of Cancer Quality of Life Core 30 (EORTC-QOL-C30) is a validated and widely used patient-reported outcome measure in cancer research and practice. It contains 30 questions that ask about activities of daily living, symptomatology, overall health and quality of life [58].

#### Guideline concordant cancer survivorship care (exploratory outcome).

We will use participant self-report to up-to-date status for of age and sex appropriate cancer screening (breast, cervical, colorectal) consistent with U.S. Preventive Services Task Force recommended screening [59–61].

### Intervention

#### Enhanced social prescribing.

We engaged adult cancer survivors to collaboratively adapt an intervention, building on the social prescribing model and using the ADAPT-ITT framework [36]. The Enhanced Social Prescribing intervention builds on the social prescribing model and also includes psychosocial educational support and is delivered in a group setting.

The purpose of the Enhanced Social Prescribing intervention is to reduce loneliness and improve social connection. The interventionist, who we call a connection navigator, will be a trained health professional (MSW). The intervention will be a virtual (audiovisual), 9-week group with 6–9 individuals.

The group will meet for an hour weekly for 9 weeks. To accommodate different processing styles, learning speeds and relational needs, the facilitator will provide an *optional* 15-minute period following the session content for further discussion and debriefing. Group meetings will include brief psychoeducation, large group discussions, breakout sessions for more intimate discussions, activities, and skills practice. In the first meeting, the navigator will start by reviewing the purpose of the group, setting group ground rules, and introducing loneliness concepts. The first activity is called “Mapping my World” where participants will think about personally important beings, places and activities in which they used to engage, currently engage and desire to engage. In the second week, the connection navigator will meet with participants individually to develop a personalized connection plan and potentially help link participants to relevant community organizations and groups. The navigator will guide each participant in developing 1–2 goals that are SMART (specific, measurable, achievable, relevant, and time-bound), and comprised of concrete steps aligned with their own priorities.

Weeks 3–8 will take place in a group format and include check-ins regarding concrete steps taken on the connections plan since the last meeting. This will include a discussion of the various dimensions of loneliness (social, emotional and existential) and what can be done to address each of these [62]. In addition, the facilitator will guide content, reflection activities, and skills practice each week relevant to themes identified during the adaptation: self-compassion, energy budgeting and pacing, intentional disclosure of the cancer experience, learning from loss and grief, asking loved ones for what you need, and advocating for your health. These topics were selected to help participants process emotions that may act as a barrier to connection, iterate on and refine their connection goals in alignment with their values, and provide skills and tools to help achieve them. Finally in week 9, participants will develop a plan to sustain the progress on their connection plans by identifying areas deserving of more attention, refining their social connection plan and developing strategies to keep this work salient in their lives.

### Enhanced usual care

Those randomized to the usual care will receive usual care through their primary care provider. They will also be emailed or mailed a list of publicly available social support resources within 2 weeks of enrollment. This list of resources mimics a clinical provider giving patients handouts or resources in clinic. The email or mailed information sheet will include a description of loneliness and its impact. We will review the resources with our adaptation advisory groups.

### Procedure

#### Data collection and data management.

**Fig 1:**
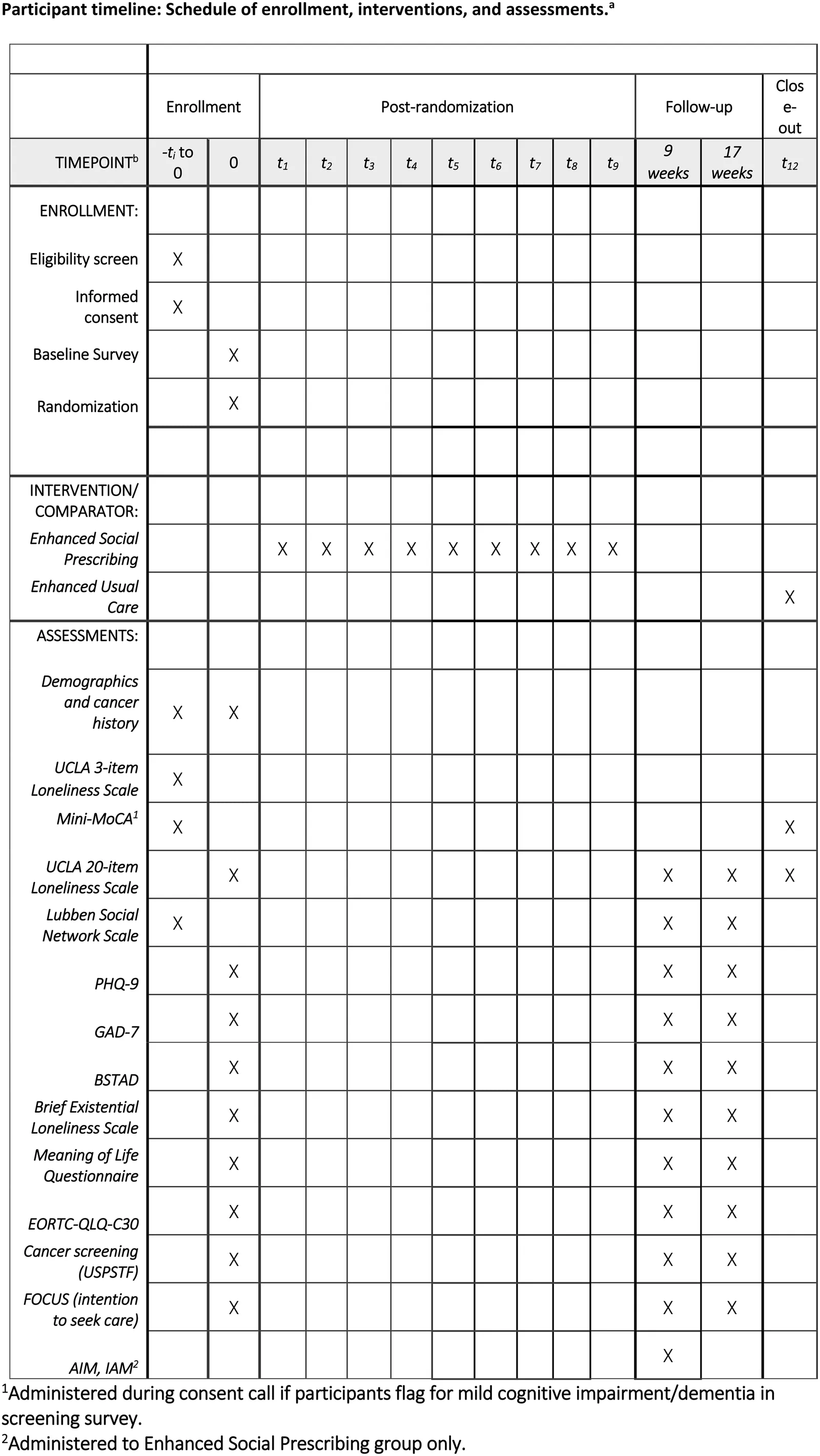
Schedule of enrollment, interventions, and assessments.

**Fig 2:**
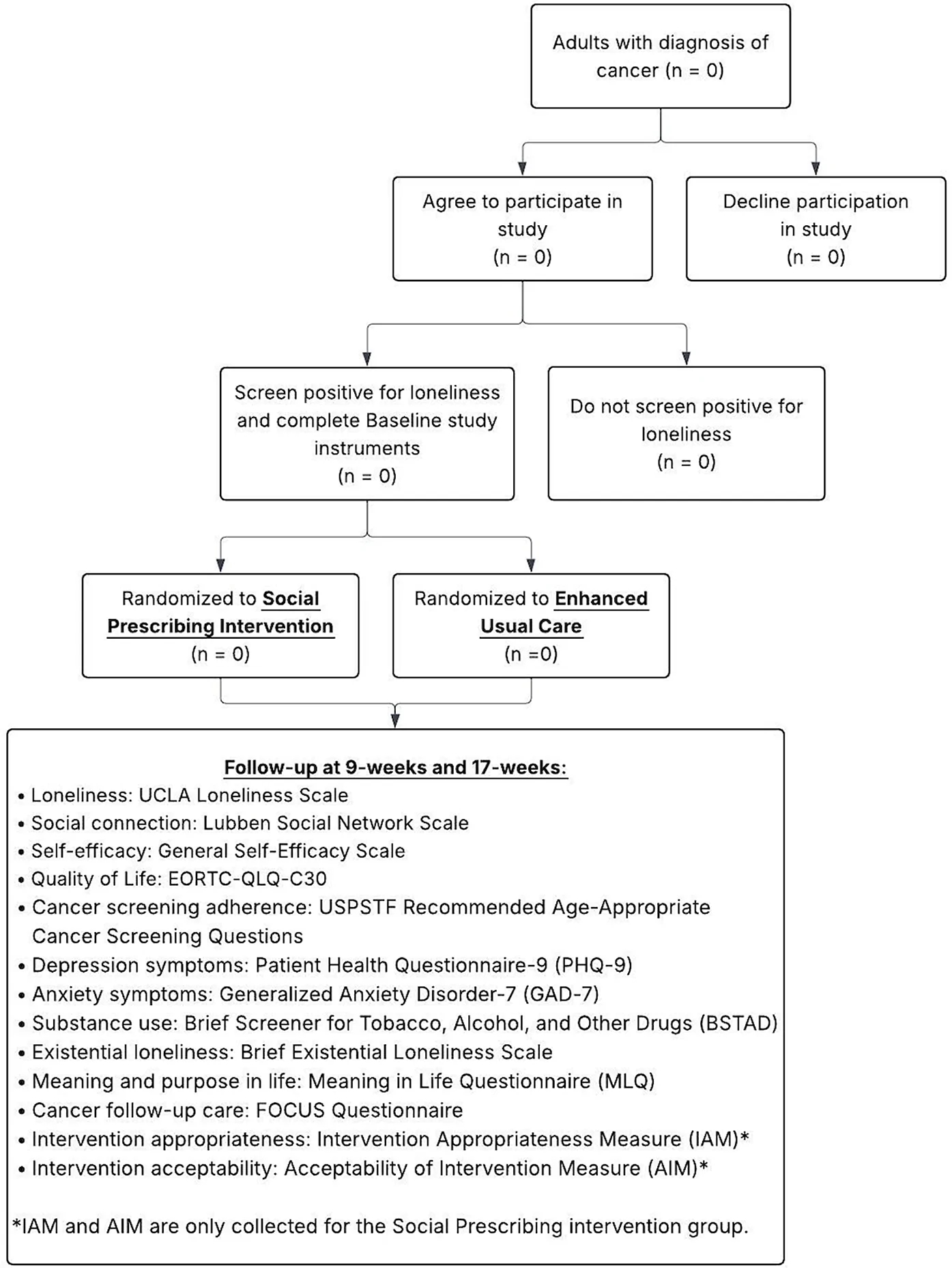
Study consort flow diagram.

All enrollment and data collection procedures as shown in Fig 1. The overall study flow in shown in Fig 2. After completing an eligible screening survey, participants will be scheduled for an informed consent call with a research team member. Upon call completion, participants will be sent a link to a REDCap the baseline survey that includes informed consent (only baseline), a demographic survey (only baseline), the UCLA Loneliness Scale (Version 3) with 20-items [48,63], Brief Scale of Existential Loneliness Scale [55], Lubben Social Network Scale- 6 item [56], Generalized Self Efficacy Scale [57], quality of life questions [64] and questions about receipt of guideline concordant cancer survivorship care [65].

We will collect the outcome measures from patients at 9 and 17 weeks after enrollment via REDCap survey. The research coordinator will conduct additional outreach, including telephone, text message, email, and written communication according to each participants’ preference to ensure complete data collection. Participants will receive USD 25 for completing each outcome assessment. Participants will be analyzed using an intent to treat analysis and included in primary outcomes analysis even if baseline data are missing. Missing data will be described to assess the amount, patterns, and reasons for missigness. Any variable with substantial missigness will undergo careful review before being considered as a candidate variable for future trials.

#### Trial monitoring.

The PI (Sebastian Tong) is responsible for monitoring trial implementation and quality. The full team will meet weekly to review study procedures, data collection, and any potential challenges. The PI will be available for urgent or time sensitive trial quality issues by email and telephone. Co-Investigators will rotate on-call coverage for after hours emergencies.

### Data analysis

#### Types of data.

All study data will be collected directly from study participants using self-report.

### Statistical analyses

#### Feasibility.

A primary goal of this study is to assess feasibility of conducting a larger-scale study to test the effectiveness of the adapted, enhanced social prescribing intervention. We will use descriptive statistics to measure study procedure and intervention feasibility outcomes.

#### Effectiveness.

This feasibility study is not powered to establish effectiveness of the intervention. However, demonstration of the feasibility and completeness of data collection is important for future research. The analysis will encompass all randomized patients. Patients will be analyzed according to their assigned intervention group, regardless of what treatment they receive (intent to treat analysis). Descriptive statistics (mean and standard deviation) will summarize the distribution of primary (loneliness), secondary (social connection, self-efficacy, quality of life) outcomes at baseline, post-intervention, and 2-month follow up. Linear effects mixed models will be used to estimate differences between the intervention arm and control arm, and changes in outcomes over the study period. The model will include the treatment arm, post-treatment time point, and their interaction as independent variables and will stratify by clinical site. Intervention assignment is modeled as a fixed effect, and participants are included as a random effect with a random intercept to account for correlation of repeated outcome measurements within individuals.

#### Reach.

We will use descriptive statistics to summarize the proportion of patients contacted who are eligible, ineligible, and agree to participate in the study. We will report the characteristics of eligible and ineligible patients by age, sex, race/ethnicity, rurality, type of primary cancer, years since cancer diagnosis and household income. These analyses are intended to identify potential trends and inform future, adequately powered studies rather than to test hypotheses.

#### Implementation.

We will use descriptive statistics to summarize participant engagement, completion of activities and attendance in sessions and then explore if these co-variates are associated with clinical outcomes. Adequate demonstration of engagement/retention in the intervention and study will be demonstrated if at least 70% of participants complete six of the nine intervention sessions, and if at least 90% of participants complete all data collection points [66]. Furthermore, we will use Fisher’s exact test to explore if there are differences in adherence/completion of the intervention based on age, sex, race/ethnicity, rurality, type of primary cancer, years since cancer diagnosis and household income. We will use descriptive statistics to summarize the fidelity to checklists completed by study interventionist as well as fidelity to protocol training.

#### Appropriateness and acceptability.

We will use descriptive statistics to summarize the acceptability and appropriateness of the intervention using the AIM and IAM measures with intervention participants. Feasibility will be explored qualitatively in interviews with the connection navigator and primary care clinical partners.

### Data management and storage

All quantitative data will be collected and stored in REDCap [67,68], hosted by the Institute of Translational Health Sciences at the University of Washington. Data cleaning, coding, and analysis will be conducted in Stata Version 19 (College Station, TX). Detailed coding files, data dictionaries and analysis programming will be stored in a secure file on the University of Washington SharePoint server. Final de-identified datasets and associated data dictionaries will be uploaded to an appropriate data repository, discoverable via persistent unique identifier. Access will be granted upon appropriate request. All data management activities will be compliant with federal, state, and local regulations and institutional human subjects division protections. No data monitoring committee is indicated for this small, feasibility study.

### Anticipated risks and benefits

Enhanced Social Prescribing may reduce loneliness and improve social connection among intervention participants. Enhanced usual care may reduce loneliness and improve social connection among participants receiving it. We will gather information from usual care participants to determine if and how they sought social support to assess this potential. Some psychological distress may result from the Enhanced Social Prescribing intervention since participants will be discussing their experiences with loneliness in the context of how to overcome loneliness. When psychological distress occurs, the study interventionist will try to address this in the context of the current session. If this is ongoing by the end of the session, the participant will be referred to resources as additional supports to address this psychological distress. If exacerbated psychological distress occurs (including suicidal ideation), the study interventionist will immediately attempt to contact the patient’s primary care clinician. If they are unavailable, the interventionist will contact one of the clinician investigators (Drs. Cole or Tong) who will assess the patient and make appropriate referrals for immediate care, including the possibility of referring the patient to the national crisis lifeline or advising them to care immediately in the emergency department.

Adverse events will be reported in accordance with local, state, and federal regulations and University of Washington Human Subjects Division policies.

### Criteria for discontinuation

The trial will be discontinued if safety monitoring identifies an unacceptable level of psychological distress among participants as defined a priori using a validated measurement instrument (e.g., PHQ-9) and reviewed by the research team.

**Patient and public involvement** – We conducted key informant interviews with patient partners and clinical staff (e.g., psychologists, social workers, oncologists) that work with cancer survivors to design the enhanced social navigation intervention and outcomes assessment. Patient partners will remain involved as key advisors throughout the project and contribute to interpretation of our results and dissemination.

### Trial governance

All scientific and programmatic decisions are made by the principal investigator and do not represent the views of the funders. This study was reviewed and approved by the University of Washington Human Subjects Division (IRB ID: STUDY00023577). Protocol updates and modifications will be submitted for review to the UWHSD.

### Dissemination

We plan to disseminate our findings in journals and at cancer control/survivorship, behavioral health, and primary care health national conferences. We will also develop a 1-page summary to disseminate the results of the study back to participating clinics and community organizations, and to other interested clinics within the WWAMI region Practice and Research Network. Demonstration of a pilot efficacy and feasibility of the Enhanced Social Prescribing intervention is essential for development of a future type 1 hybrid effectiveness-implementation trial that tests the Enhanced Social Prescribing intervention for cancer survivors with loneliness on a larger scale. The Enhanced Social Prescribing intervention has the potential to have substantial impact by reducing loneliness in a high-risk population (adult cancer survivors with loneliness) who already face substantial physical and mental health disparities. This proposal not only advances care for adult cancer survivors but also has the potential to have substantial impact in building community-clinical partnerships to address other disparities and improve overall community health and mind-body-spirit well-being.

## Discussion

### Expected findings

We anticipate successful recruitment of 36 adult cancer survivors with loneliness, delivery of the Enhanced Social Prescribing intervention to 18 participants and delivery of enhanced usual care to 18 participants. We anticipate high study retention, with at least 70% of participants will completing all data collection and high intervention engagement with at least 70% of participants assigned to the intervention attending at least 2/3 of the intervention visits. This feasibility study will demonstrate the feasibility of conducting a larger-scale randomized controlled trial to test the effectiveness and study the implementation of the Enhanced Social Prescribing intervention. While not powered for statistical significance, we also anticipate that the Enhanced Social Prescribing intervention will lead to reduced loneliness among adult cancer survivors as measured by the UCLA Loneliness Scale.

### Limitations

Several limitations exist. First, participant sample bias might occur, since the participants who agree to participate in this study may be more motivated to address loneliness than the general population. However, this is likely to bias us toward the null, given our plan for randomization. Second, this feasibility study is not intended to establish effectiveness of the intervention, and sub-group analyses are not possible with this small sample.

## Conclusions

Loneliness in cancer survivors affects well-being, health outcomes and concordance with guideline recommended care. Cancer survivors have specialized needs for addressing loneliness given unique contributors to loneliness and life experiences in this population. To our knowledge, our study is the first to explore using an adapted social prescribing intervention to address loneliness in cancer survivors and has the potential to substantially improve the health and well-being of cancer survivors.

## Supporting information

S1 Checklist S1 SPIRIT 2025 editable checklist. (DOCX)
