# Supplementary material for: An adapted enhanced social prescribing intervention vs. usual care in the treatment of loneliness in cancer survivors: Study protocol for a feasibility pilot randomized controlled trial
Source: PLoS One. 2026 Sep 18;21(9):e0350956. doi: 10.1371/journal.pone.0350956 (PMC13588344; doi:10.1371/journal.pone.0350956)
Supplement: Supplementary file 3 [file pone.0350956.s004.docx]

August 24, 2026

Dear Editor,

We are pleased to submit our updated revised manuscript “An Adapted Enhanced Social Prescribing Intervention vs. Usual Care in the Treatment of Loneliness in Cancer Survivors: Study Protocol to Assess Feasibility of a Randomized Controlled Trial.” We have carefully reviewed the remaining comments from reviewers as well as the guidance from the editor. Table 1 summarizes the most recent changes made in response to the suggestions. Table 2 summarizes the changes made in response to the first revison.

Sincerely,

Allison Cole, MD, MPH
[acole2@uw.edu](mailto:acole2@uw.edu)

206-909-2380

| Table 1: Response to second review | | | |
| --- | --- | --- | --- |
| **Reviewer 1: Not applicable** |  |  |  |
| **Reviewer 2** |  |  |  |
| The new progression table needs number | Table numbering has been added. The table is now cited in the text. | Outcomes, page 12. |  |
| Concern that progression “Red” for 3+ participant drop out by 3 months. | We have updated the “Red” criteria to be “not applicable” with a footnote explaining that if drop out exceeds 2, we will undertake design review. | Outcomes pages 12-13 |  |
| Sample size is too small for MICE analysis of missing data. | We have changed the missing data analysis to be descriptive and informative of future trials. | Procedure, pages 17-18 |  |
| Make plans for making data from the feasibility study available | We have indicated that feasibility assessment data will be available on appropriate request. | Title page, page 2 |  |

| **Table 2: Response to reviewers from First Review** | | |
| --- | --- | --- |
| **Reviewer 1** | Response | Location |
| Replace “background” with “Introduction” | This change has been made | Abstract  Page 5 |
| Place aim of the study under “Introduction” at the end of the introduction section. | This change has been made | Pages 6-7 |
| Change to passive writing style |  |  |
| Include clinical trial protocol registration | NCT registration is reported in the abstract. This additiona is now also in the methods | Page 7 |
| How were primary care practices  selected from among the 140. Provide more detail on the sampling process. | Additional detail has been added: To ensure participation from rural cancer survivors, we elected to invite all rural and rural-serving primary care practices in the WPRN to consider participating. Invitations to participate were shared electronically with champions (physician representatives to the WPRN) from each eligible rural or rural-serving health system (n=19). Champions were instructed to respond by email with interest. The study PI and research coordinator met with interested champions (4) to share more details about the study and affirm interest/eligibility. Three interested sites affirmed willingness and capacity to support recruitment and two enrolled in the study. | Page 8 |
| Provide rationale for selecting only two clinics | Text has been added: We limited participation to two sites based on anticipated sample size and feasibility. | Page 8 |
| Performing a sample size calculation is encouraged. Clustering effects should be considered when recruiting participants from multiple sites or practices, as intracluster correlation may increase the required sample size. | While we agree that sample size calculations are important for clinical trials, the goal of this study is to demonstrate feasibility of recruitment and intervention delivery procedures and gather pilot data about the primary outcomes means and ranges, which will be essential in future sample size and power calculations. | Page 8 |
| Replace >= with single symbol | This change has been made | Page 8 |
| P9, line 20: Randomization will be conducted using REDCap, and procedures to ensure allocation concealment is to be stated. | This statement has been added: Randomization will be conducted using REDCap. Procedures to ensure allocation concealment include using parallel structures in REDcap for each arm, which reduces the possibility that study personnel interacting with the database can infer treatment assignment. | Page 10 |
| P10, line 18: How many clinics will be involved? Location? More information is required. | Additional information about sites has been added. We are not able to name or provide the specific location of sites to ensure their privacy. | Page 10. |
| $15 replaced with USD15 | This change has been made | Page 11 |
| With 36 participants, the study may be underpowered to generate definitive outcome estimates and is more appropriate for ‘feasibililty/exploratory’ analysis | We have changed the goals of the study throughout to reflect the feasibility nature of the study. |  |
| A large number of questionnaires and inventories have been proposed for this study, which may pose a burden to participants. Completing all instruments may be demanding and potentially exhausting. Will participants receive compensation or a token of appreciation for their participation in the study? | Individuals receive USD 25 for each outcome assessment completed. This information has been added. | P 17 |
| The use of multiple imputation is not guaranteed as its appropriateness depends on several assumptions and methodological caveats that must be carefully considered. | We have added language to reflect this possibility. | Page 17 |
| P17 Line 1: PI (Sebastian Tong) | This change has been made | Page 17 |
| P17 Line 20: Linear mixed effects models | This change has been made | Page 18 |
| P 17, lines 23-24:  The sentence could be improved e.g. Intervention assignment is modelled as a fixed effect and participants are included as a random effect with a random intercept to account for correlation of repeated outcome measurements within individuals. | This change has been made | Page 18 |
| P18, line 3 & 13: How certain Fisher’s exact test will be used | We have removed reference to Fisher’s exact test in the Reach analysis.  We have clarified that Fisher’s exact test will be used to compare intervention completion by participant characteristics (e.g., sex) | Page 18 |
| P20 Line 1-2: he sentence could be improved e.g. The trial will be discontinued if safety monitoring identifies an unacceptable level of psychological distress among participants as defined a priori using a validated measurement instrument and reviewed by the data monitoring committee. | This change has been made. | Page 20 |
| P20 Line 6: reference number to be added | This change has been made | Page 20 |
| Term ‘pilot’ prelaced with feasibility when the study is designed/reported as a feasibility study | We have made this change throughout the paper. |  |
| Use bracket style text for references | This change has been made |  |
| **Reviewer 2:** |  |  |
| The protocol is a bit inconsistent about what kind of study this is. It states the trial is not powered for effectiveness, yet it says loneliness as the and plans linear mixed models and includes an arm by time interaction with site stratification and a participant random effect. With only 36 participants total (18 per arm), this model is over-parameterised and the between-arm estimates will likely be too imprecise to be informative. I would encourage the authors to reframe in line with the CONSORT extension for pilot and feasibility trials. The manuscript can be strengthened if the focus/primary objectives are feasibility and process parameters, and the outcome measures like loneliness used to estimate the parameters needed to design the main trial, not to test for an effect. So I would suggest describing loneliness etc as the candidate primary outcome for a future trial rather than for this study. Reporting feasibility estimates with CIs, and clinical outcomes descriptively, would be stronger than the proposed model. | We have revised the manuscript to reflect the feasibility design and goals and to reduce the emphasis on efficacy. |  |
| No sample size calculation was performed because this is a pilot but the choice of 18 per arm still needs a stated rationale. Please justify the number with reference to the pilot sample size literature. As with point above, being clearer about that the is intended to deliver, e.g. estimate of the recruitment, retention and SD of the future trial candidate primary outcome. | A rationale for the sample size is now provided. 36 participants was selected as an enrollment goal based on ensuring sufficient enrollment for at least two cohorts for the enhanced social prescribing intervention and matching enrollment to the available resources and timeline. | Page 8 |
| Random permutated blocks are mentioned but not details (block size, stratification etc). Please provide more details. Also the allocation concealment mechanism. |  |  |
| For a complex social intervention like this, the control condition and contamination are central feasibility questions at this stage and at the moment this is underdeveloped. The brochure is described briefly and would benefit from TIDieR-level detail (maybe this could be in supplement?) . The protocol does not clearly say how the authors will measure the control arm experience: attrition, and whether control participants independently sought social support, attended groups etc. Without this, contamination and the true contrast between arms cannot be assessed in the pilot or designed for in the main trial. Again maybe add to the supplement to save on words in the main text. | Because the enhanced social prescribing groups (intervention) are provided only to enrolled participants, held virtually, and not open to the public, we do not anticipate any potential contamination between treatment arms. We have clarified that all measures will be collected at the same time points by the same process for both intervention and control patients. We have also described that control patients will be asked about any activities they may have engaged in to obtain social support.  The enhanced usual care description has been edited to more accurately reflect usual care. | Page 19, Page 16 |
| I was unclear exactly how adherence/engagement assessed at 6 and 9 weeks | Adherence and engagement will be determined based on review of notes and data gathered by the intervention facilitator during the course of the intervention. This information is included in the manuscript. | Page 12-13 |
| Does the group delivery for control and individual for intervention introduce some issues that need to be discussed which may have implications for main trial modelling? i.e. group-level clustering. Could estimating group level ICC be useful? | The control condition does not involve group delivery. We have made this clearer in the manuscript. |  |
| PPI are NA but earlier sections seems like you did lots to develop this work to co-adapt and help of patient groups? This is really great and I think you can complete this section as it seems like there was input in the design, materials etc. | We have added detail about this: We conducted key informant interviews with patient partners and clinical staff (e.g., psychologists, social workers, oncologists) that work with cancer survivors to design the enhanced social navigation intervention and outcomes assessment. Patient partners will remain involved as key advisors throughout the project and contribute to interpretation of our results and dissemination. | Page 20 |
| Overall I think you can make the progression criteria clearer, e.g. using traffic light scheme. Maybe bring these together into a single progression table (again supplement if not main text) | A table with progression criteria has been added. | Page 12 |
| Some of the patient reported outcomes do not appear to be listed in Fig 1? Eg self efficacy, and some appear to be in the table but not the text. Please check and make sure inclusion of all is consistent. |  |  |
| Eligibility is based on 3-item UCLA scale, while the outcome is the 20-item UCLA scale. I wonder if selecting participants on elevated loneliness and then re-measuring will produce apparent improvement through regression to the mean, which is relevant to how any within-group change is interpreted. Using a different instrument for screening and outcome may mitigate partly. Please clarify whether the longer scale is also captured at baseline. Fig 1 suggests it is but I was unsure from the text. | Initial screening for enrollment is done using the 3-item scale. All enrolled participants will complete the full 20-item UCLA scale at baseline and outcome assessments and the change full scale will be used for outcome analysis. | Page 17 |
| Typo? Page 10 refers to the two intervention arms | This has been corrected. |  |
| Unclear what year of SPIRIT. Appears to be more recent version but text cites earlier version. | We reference the SPIRIT 2013 and 2025me. | Page 3 |
| My view is that applying MICE for loss to follow-up and to include participants with missing baseline data in an ITT analysis is not appropriate for a pilot study. Describing the pattern and extent of missingness is the more useful. | We have updated the analysis to reflect this recommendation. | Page 17 |
| The discontinuation rule about distress could do with a clearer definition, e.g. what threshold, over what time period etc | This has been edited. | Page 20 |
|  |  |  |
