# Supplementary material for: An adapted enhanced social prescribing intervention vs. usual care in the treatment of loneliness in cancer survivors: Study protocol for a feasibility pilot randomized controlled trial
Source: PLoS One. 2026 Sep 18;21(9):e0350956. doi: 10.1371/journal.pone.0350956 (PMC13588344; doi:10.1371/journal.pone.0350956)
Supplement: Peer review history

# Peer review: Decision Letter 0

**DOI:** 10.1371/journal.pone.0350956.r001

**Authors:** Johanna Pruller

22 Jun 2026

Dear Dr. Cole,

Thank you for submitting your manuscript to PLOS ONE. After careful consideration, we feel that it has merit but does not fully meet PLOS ONE’s publication criteria as it currently stands. Therefore, we invite you to submit a revised version of the manuscript that addresses the points raised during the review process.

Please submit your revised manuscript by Aug 06 2026 11:59PM. If you will need more time than this to complete your revisions, please reply to this message or contact the journal office at plosone@plos.org. When you’re ready to submit your revision, log on to https://www.editorialmanager.com/pone/ and select the ‘Submissions Needing Revision’ folder to locate your manuscript file.

- A letter that responds to each point raised by the academic editor and reviewer(s). You should upload this letter as a separate file labeled ‘Response to Reviewers’.
- A marked-up copy of your manuscript that highlights changes made to the original version. You should upload this as a separate file labeled ‘Revised Manuscript with Track Changes’.
- An unmarked version of your revised paper without tracked changes. You should upload this as a separate file labeled ‘Manuscript’.

If you would like to make changes to your financial disclosure, please include your updated statement in your cover letter. Guidelines for resubmitting your figure files are available below the reviewer comments at the end of this letter.

If applicable, we recommend that you deposit your laboratory protocols in protocols.io to enhance the reproducibility of your results. Protocols.io assigns your protocol its own identifier (DOI) so that it can be cited independently in the future. For instructions see: https://journals.plos.org/plosone/s/submission-guidelines#loc-laboratory-protocols. Additionally, PLOS ONE offers an option for publishing peer-reviewed Lab Protocol articles, which describe protocols hosted on protocols.io. Read more information on sharing protocols at https://plos.org/protocols?utm_medium=editorial-email&utm_source=authorletters&utm_campaign=protocols.

As the corresponding author, your ORCID iD is verified in the submission system and will appear in the published article. PLOS supports the use of ORCID, and we encourage all coauthors to register for an ORCID iD and use it as well. Please encourage your coauthors to verify their ORCID iD within the submission system before final acceptance, as unverified ORCID iDs will not appear in the published article. *Only*  the individual author can complete the verification step; PLOS staff *cannot*  verify ORCID iDs on behalf of authors.

We look forward to receiving your revised manuscript.

Kind regards,

Johanna Pruller, Ph.D.

Senior Editor

PLOS One

Journal Requirements:

When submitting your revision, we need you to address these additional requirements.

1.Please ensure that your manuscript meets PLOS ONE’s style requirements, including those for file naming. The PLOS ONE style templates can be found at

https://journals.plos.org/plosone/s/file?id=wjVg/PLOSOne_formatting_sample_main_body.pdf and

https://journals.plos.org/plosone/s/file?id=ba62/PLOSOne_formatting_sample_title_authors_affiliations.pdf

2. Please note that funding information should not appear in any section or other areas of your manuscript. We will only publish funding information present in the Funding Statement section of the online submission form. Please remove any funding-related text from the manuscript.

3. We note that the grant information you provided in the ‘Funding Information’ and ‘Financial Disclosure’ sections do not match.

When you resubmit, please ensure that you provide the correct grant numbers for the awards you received for your study in the ‘Funding Information’ section.

4. Thank you for stating the following financial disclosure:

“Hutchinson Center for Cancer Outcomes Research at the Fred Hutch Cancer Center Trials Expansion Program”

Please state what role the funders took in the study.  If the funders had no role, please state: "The funders had no role in study design, data collection and analysis, decision to publish, or preparation of the manuscript."

If this statement is not correct you must amend it as needed.

Please include this amended Role of Funder statement in your cover letter; we will change the online submission form on your behalf.

5. Your ethics statement should only appear in the Methods section of your manuscript. If your ethics statement is written in any section besides the Methods, please delete it from any other section.

6. Please include a caption for figure 1.

7. Please include captions for your Supporting Information files at the end of your manuscript, and update any in-text citations to match accordingly. Please see our Supporting Information guidelines for more information: http://journals.plos.org/plosone/s/supporting-information.

8. Please upload a copy of your study protocol that was approved by your ethics committee/IRB as a Supporting Information file. By the study protocol, we mean the complete and detailed plan for the conduct and analysis of the trial approved by the ethics committee/IRB. Please send this in the original language. If this is in a language other than English, please also provide a translation. [https://journals.plos.org/plosone/s/submission-guidelines#loc-guidelines-for-specific-study-types

9. If the reviewer comments include a recommendation to cite specific previously published works, please review and evaluate these publications to determine whether they are relevant and should be cited. There is no requirement to cite these works unless the editor has indicated otherwise.

Additional Editor Comments:

The manuscript has been evaluated by two reviewers, and their comments are available below.

The reviewers have raised a number of concerns that need attention. Could you please revise the manuscript to carefully address the concerns raised?

[Note: HTML markup is below. Please do not edit.]

Reviewers’ comments:

Reviewer’s Responses to Questions

**Comments to the Author**

1. Does the manuscript provide a valid rationale for the proposed study, with clearly identified and justified research questions?

Reviewer #1: Partly

Reviewer #2: Yes

**********

2. Is the protocol technically sound and planned in a manner that will lead to a meaningful outcome and allow testing the stated hypotheses?

Reviewer #1: Partly

Reviewer #2: Partly

**********

3. Is the methodology feasible and described in sufficient detail to allow the work to be replicable?

Reviewer #1: Yes

Reviewer #2: No

**********

4. Have the authors described where all data underlying the findings will be made available when the study is complete?

The PLOS Data policy

Reviewer #1: Yes

Reviewer #2: No

**********

5. Is the manuscript presented in an intelligible fashion and written in standard English?

Reviewer #1: Yes

Reviewer #2: Yes

**********

Please use the space provided to explain your answers to the questions above and, if applicable, provide comments about issues authors must address before this protocol can be accepted for publication. You may also include additional comments for the author, including concerns about research or publication ethics.

You may also provide optional suggestions and comments to authors that they might find helpful in planning their study.

Reviewer #1: Line 1: Subtitle ‘Background’ is to be replaced with ‘Introduction’.

Page 7 Line 1: The aim of the study is to be placed under Introduction at the end of introduction section.

Page 7: Excessive repetition of the word ‘we’ was observed. A passive writing style is preferred and the use of first-person pronouns could be minimized/omitted.

Page 7, Line 14: The clinical trial protocol registration is to be provided.

Page 8 :Line 5: It is unclear how the primary practices were selected from 140 primary practices. More information on the sampling process is to be provided.

Page 8 Line 9: The rationale for selecting only two clinics and not more than 2 should be clearly justified in the study protocol, especially when 5-state regions are involved.

Page 8 Line 13: Unless the study is solely intended to assess feasibility, performing a sample size calculation is generally encouraged even for pilot studies. In addition, clustering effects should be considered when recruiting participants from multiple sites or practices, as intracluster correlation may increase the required sample size.

Page 8 Line 17: Symbol ‘>= ‘ is to be replaced with ≥

Page 9 Line 20: Randomization will be conducted using REDCap, and procedures to ensure allocation concealment is to be stated.

Page 10 Line 18: How many clinics will be involved? Location? More information is required.

Page 11 Line 7: $15 to be replaced with USD15.

Page 11 Line 13: With 36 participants (n = 18 per group), the study may be underpowered to generate definitive outcome estimates and is more appropriate for ‘feasibility’/‘exploratory analysis’.

A large number of questionnaires and inventories have been proposed for this study, which may pose a burden to participants. Completing all instruments may be demanding and potentially exhausting. Will participants receive compensation or a token of appreciation for their participation in the study?

Page 16, Line 20: The use of multiple imputation is not guaranteed as its appropriateness depends on several assumptions and methodological caveats that must be carefully considered.

Page 17 Line 1: PI (Sebastian Tong)

Page 17 Line 20: Linear mixed-effects models

Page 17 Line 23-24: The sentence could be improved e.g. Intervention assignment is modelled as a fixed effect and participants are included as a random effect with a random intercept to account for correlation of repeated outcome measurements within individuals.

Page 18 Line 3 & 13: How certain Fisher’s exact test will be used?

Page 20 Line 1-2: Trials are usually stopped based on predefined stopping rules, Data and Safety Monitoring Board (DSMB) review or serious adverse events and not just percentage thresholds alone. The sentence could be improved e.g. The trial will be discontinued if safety monitoring identifies an unacceptable level of psychological distress among participants as defined a priori using a validated measurement instrument and reviewed by the data monitoring committee.

Page 20 Line 6: Reference number is to be added.

The term ‘pilot’ may be replaced with ‘feasibility’ when the study is designed/reported as a feasibility study.

Ensure that in-text citations are formatted in bracket style and not in superscript format.

Reviewer #2: Overall this is a nicely written protocol addressing an important and under-served problem. Loneliness in cancer survivors is an important topic, the adaptation work is a real strength, as is the implementation focus. I am interested to see the results when the pilot is completed. My comments are intended to help the authors sharpen the framing of the study as a pilot/feasibility trial and to resolve several internal inconsistencies. I think they are addressable in revision

1. I feel the protocol is a bit inconsistent about what kind of study this is. It states the trial is not powered for effectiveness, yet it says loneliness as the and plans linear mixed models and includes an arm by time interaction with site stratification and a participant random effect. With only 36 participants total (18 per arm), this model is over-parameterised and the between-arm estimates will likely be too imprecise to be informative. I would encourage the authors to reframe in line with the CONSORT extension for pilot and feasibility trials. The manuscript can be strengthened if the focus/primary objectives are feasibility and process parameters, and the outcome measures like loneliness used to estimate the parameters needed to design the main trial, not to test for an effect. So I would suggest describing loneliness etc as the candidate primary outcome for a future trial rather than for this study. Reporting feasibility estimates with CIs, and clinical outcomes descriptively, would be stronger than the proposed model.

2. no sample size calculation was performed because this is a pilot but the choice of 18 per arm still needs a stated rationale. Please justify the number with reference to the pilot sample size literature. As with point above, being clearer about that the is intended to deliver, e.g. estimate of the recruitment, retention and SD of the future trial candidate primary outcome.

3. Random permutated blocks are mentioned but not details (block size, stratification etc). Please provide more details. Also the allocation concealment mechanism.

4. For a complex social intervention like this, the control condition and contamination are central feasibility questions at this stage and at the moment this is underdeveloped. The brochure is described briefly and would benefit from TIDieR-level detail (maybe this could be in supplement?) . The protocol does not clearly say how the authors will measure the control arm experience: attrition, and whether control participants independently sought social support, attended groups etc. Without this, contamination and the true contrast between arms cannot be assessed in the pilot or designed for in the main trial. Again maybe add to the supplement to save on words in the main text.

5. I was unclear exactly how adherence/engagement assessed at 6 and 9 weeks

6. Does the group delivery for control and individual for intervention introduce some issues that need to be discussed which may have implications for main trial modelling? i.e. group-level clustering. Could estimating group level ICC be useful?

7. PPI are NA but earlier sections seems like you did lots to develop this work to co-adapt and help of patient groups? This is really great and I think you can complete this section as it seems like there was input in the design, materials etc.

8. Overall I think you can make the progression criteria clearer, e.g. using traffic light scheme. Maybe bring these together into a single progression table (again supplement if not main text)

9. Some of the patient reported outcomes do not appear to be listed in Fig 1? Eg self efficacy, and some appear to be in the table but not the text. Please check and make sure inclusion of all is consistent.

10. Eligibility is based on 3-item UCLA scale, while the outcome is the 20-item UCLA scale. I wonder if selecting participants on elevated loneliness and then re-measuring will produce apparent improvement through regression to the mean, which is relevant to how any within-group change is interpreted. Using a different instrument for screening and outcome may mitigate partly. Please clarify whether the longer scale is also captured at baseline. Fig 1 suggests it is but I was unsure from the text.

Minor

10. Typo? Page 10 refers to the two intervention arms

11. Unclear what year of SPIRIT. Appears to be more recent version but text cites earlier version.

12. My view is that applying MICE for loss to follow-up and to include participants with missing baseline data in an ITT analysis is not appropriate for a pilot study. Describing the pattern and extent of missingness is the more useful.

13. The discontinuation rule about distress could do with a clearer definition, e.g. what threshold, over what time period etc

**********

what does this mean?). If published, this will include your full peer review and any attached files.

If you choose “no”, your identity will remain anonymous but your review may still be made public.

**Do you want your identity to be public for this peer review?**  For information about this choice, including consent withdrawal, please see our Privacy Policy

Reviewer #1: No

Reviewer #2: **Yes:** Jenny Harris

**********

[NOTE: If reviewer comments were submitted as an attachment file, they will be attached to this email and accessible via the submission site. Please log into your account, locate the manuscript record, and check for the action link "View Attachments". If this link does not appear, there are no attachment files.]

To ensure your figures meet our technical requirements, please review our figure guidelines: https://journals.plos.org/plosone/s/figures

You may also use PLOS’s free figure tool, NAAS, to help you prepare publication quality figures: https://journals.plos.org/plosone/s/figures#loc-tools-for-figure-preparation.

NAAS will assess whether your figures meet our technical requirements by comparing each figure against our figure specifications.

# Author comment: Author response to Decision Letter 1

**DOI:** 10.1371/journal.pone.0350956.r002

31 Jul 2026

Reviewer 1 Response Location

Replace “background” with “Introduction” This change has been made Abstract

Page 5

Place aim of the study under “Introduction” at the end of the introduction section. This change has been made Pages 6-7

Change to passive writing style

Include clinical trial protocol registration NCT registration is reported in the abstract. This additiona is now also in the methods Page 7

How were primary care practices selected from among the 140. Provide more detail on the sampling process. Additional detail has been added: To ensure participation from rural cancer survivors, we elected to invite all rural and rural-serving primary care practices in the WPRN to consider participating. Invitations to participate were shared electronically with champions (physician representatives to the WPRN) from each eligible rural or rural-serving health system (n=19). Champions were instructed to respond by email with interest. The study PI and research coordinator met with interested champions (4) to share more details about the study and affirm interest/eligibility. Three interested sites affirmed willingness and capacity to support recruitment and two enrolled in the study. Page 8

Provide rationale for selecting only two clinics Text has been added: We limited participation to two sites based on anticipated sample size and feasibility. Page 8

Performing a sample size calculation is encouraged. Clustering effects should be considered when recruiting participants from multiple sites or practices, as intracluster correlation may increase the required sample size. While we agree that sample size calculations are important for clinical trials, the goal of this study is to demonstrate feasibility of recruitment and intervention delivery procedures and gather pilot data about the primary outcomes means and ranges, which will be essential in future sample size and power calculations. Page 8

Replace >= with single symbol This change has been made Page 8

P9, line 20: Randomization will be conducted using REDCap, and procedures to ensure allocation concealment is to be stated. This statement has been added: Randomization will be conducted using REDCap. Procedures to ensure allocation concealment include using parallel structures in REDcap for each arm, which reduces the possibility that study personnel interacting with the database can infer treatment assignment.

Page 10

P10, line 18: How many clinics will be involved? Location? More information is required.

Additional information about sites has been added. We are not able to name or provide the specific location of sites to ensure their privacy. Page 10.

$15 replaced with USD15 This change has been made Page 11

With 36 participants, the study may be underpowered to generate definitive outcome estimates and is more appropriate for ‘feasibililty/exploratory’ analysis We have changed the goals of the study throughout to reflect the feasibility nature of the study.

A large number of questionnaires and inventories have been proposed for this study, which may pose a burden to participants. Completing all instruments may be demanding and potentially exhausting. Will participants receive compensation or a token of appreciation for their participation in the study? Individuals receive USD 25 for each outcome assessment completed. This information has been added. P 17

The use of multiple imputation is not guaranteed as its appropriateness depends on several assumptions and methodological caveats that must be carefully considered. We have added language to reflect this possibility. Page 17

P17 Line 1: PI (Sebastian Tong) This change has been made Page 17

P17 Line 20: Linear mixed effects models This change has been made Page 18

P 17, lines 23-24: The sentence could be improved e.g. Intervention assignment is modelled as a fixed effect and participants are included as a random effect with a random intercept to account for correlation of repeated outcome measurements within individuals. This change has been made Page 18

P18, line 3 & 13: How certain Fisher’s exact test will be used We have removed reference to Fisher’s exact test in the Reach analysis.

We have clarified that Fisher’s exact test will be used to compare intervention completion by participant characteristics (e.g., sex) Page 18

P20 Line 1-2: he sentence could be improved e.g. The trial will be discontinued if safety monitoring identifies an unacceptable level of psychological distress among participants as defined a priori using a validated measurement instrument and reviewed by the data monitoring committee. This change has been made. Page 20

P20 Line 6: reference number to be added This change has been made Page 20

Term ‘pilot’ prelaced with feasibility when the study is designed/reported as a feasibility study We have made this change throughout the paper.

Use bracket style text for references This change has been made

Reviewer 2:

The protocol is a bit inconsistent about what kind of study this is. It states the trial is not powered for effectiveness, yet it says loneliness as the and plans linear mixed models and includes an arm by time interaction with site stratification and a participant random effect. With only 36 participants total (18 per arm), this model is over-parameterised and the between-arm estimates will likely be too imprecise to be informative. I would encourage the authors to reframe in line with the CONSORT extension for pilot and feasibility trials. The manuscript can be strengthened if the focus/primary objectives are feasibility and process parameters, and the outcome measures like loneliness used to estimate the parameters needed to design the main trial, not to test for an effect. So I would suggest describing loneliness etc as the candidate primary outcome for a future trial rather than for this study. Reporting feasibility estimates with CIs, and clinical outcomes descriptively, would be stronger than the proposed model. We have revised the manuscript to reflect the feasibility design and goals and to reduce the emphasis on efficacy.

No sample size calculation was performed because this is a pilot but the choice of 18 per arm still needs a stated rationale. Please justify the number with reference to the pilot sample size literature. As with point above, being clearer about that the is intended to deliver, e.g. estimate of the recruitment, retention and SD of the future trial candidate primary outcome. A rationale for the sample size is now provided. 36 participants was selected as an enrollment goal based on ensuring sufficient enrollment for at least two cohorts for the enhanced social prescribing intervention and matching enrollment to the available resources and timeline. Page 8

Random permutated blocks are mentioned but not details (block size, stratification etc). Please provide more details. Also the allocation concealment mechanism.

For a complex social intervention like this, the control condition and contamination are central feasibility questions at this stage and at the moment this is underdeveloped. The brochure is described briefly and would benefit from TIDieR-level detail (maybe this could be in supplement?) . The protocol does not clearly say how the authors will measure the control arm experience: attrition, and whether control participants independently sought social support, attended groups etc. Without this, contamination and the true contrast between arms cannot be assessed in the pilot or designed for in the main trial. Again maybe add to the supplement to save on words in the main text. Because the enhanced social prescribing groups (intervention) are provided only to enrolled participants, held virtually, and not open to the public, we do not anticipate any potential contamination between treatment arms. We have clarified that all measures will be collected at the same time points by the same process for both intervention and control patients. We have also described that control patients will be asked about any activities they may have engaged in to obtain social support.

The enhanced usual care description has been edited to more accurately reflect usual care. Page 19, Page 16

I was unclear exactly how adherence/engagement assessed at 6 and 9 weeks Adherence and engagement will be determined based on review of notes and data gathered by the intervention facilitator during the course of the intervention. This information is included in the manuscript. Page 12-13

Does the group delivery for control and individual for intervention introduce some issues that need to be discussed which may have implications for main trial modelling? i.e. group-level clustering. Could estimating group level ICC be useful? The control condition does not involve group delivery. We have made this clearer in the manuscript.

PPI are NA but earlier sections seems like you did lots to develop this work to co-adapt and help of patient groups? This is really great and I think you can complete this section as it seems like there was input in the design, materials etc. We have added detail about this: We conducted key informant interviews with patient partners and clinical staff (e.g., psychologists, social workers, oncologists) that work with cancer survivors to design the enhanced social navigation intervention and outcomes assessment. Patient partners will remain involved as key advisors throughout the project and contribute to interpretation of our results and dissemination. Page 20

Overall I think you can make the progression criteria clearer, e.g. using traffic light scheme. Maybe bring these together into a single progression table (again supplement if not main text) A table with progression criteria has been added. Page 12

Some of the patient reported outcomes do not appear to be listed in Fig 1? Eg self efficacy, and some appear to be in the table but not the text. Please check and make sure inclusion of all is consistent.

Eligibility is based on 3-item UCLA scale, while the outcome is the 20-item UCLA scale. I wonder if selecting participants on elevated loneliness and then re-measuring will produce apparent improvement through regression to the mean, which is relevant to how any within-group change is interpreted. Using a different instrument for screening and outcome may mitigate partly. Please clarify whether the longer scale is also captured at baseline. Fig 1 suggests it is but I was unsure from the text. Initial screening for enrollment is done using the 3-item scale. All enrolled participants will complete the full 20-item UCLA scale at baseline and outcome assessments and the change full scale will be used for outcome analysis. Page 17

Typo? Page 10 refers to the two intervention arms This has been corrected.

Unclear what year of SPIRIT. Appears to be more recent version but text cites earlier version. We reference the SPIRIT 2013 and 2025me. Page 3

My view is that applying MICE for loss to follow-up and to include participants with missing baseline data in an ITT analysis is not appropriate for a pilot study. Describing the pattern and extent of missingness is the more useful. We have updated the analysis to reflect this recommendation. Page 17

The discontinuation rule about distress could do with a clearer definition, e.g. what threshold, over what time period etc This has been edited. Page 20

Attachment Submitted filename: FLoC Manuscript Response to Reviewers Table.docx

# Peer review: Decision Letter 1

**DOI:** 10.1371/journal.pone.0350956.r003

**Authors:** Omid Beiki

24 Aug 2026

Dear Dr. Cole,

Thank you for submitting your manuscript to PLOS One. After careful consideration, we feel that it has merit but does not fully meet PLOS One’s publication criteria as it currently stands. Therefore, we invite you to submit a revised version of the manuscript that addresses the points raised during the review process.

Please submit your revised manuscript by Oct 08 2026 11:59PM. If you will need more time than this to complete your revisions, please reply to this message or contact the journal office at plosone@plos.org. When you’re ready to submit your revision, log on to https://www.editorialmanager.com/pone/ and select the ‘Submissions Needing Revision’ folder to locate your manuscript file.

- A letter that responds to each point raised by the academic editor and reviewer(s). You should upload this letter as a separate file labeled ‘Response to Reviewers’.
- A marked-up copy of your manuscript that highlights changes made to the original version. You should upload this as a separate file labeled ‘Revised Manuscript with Track Changes’.
- An unmarked version of your revised paper without tracked changes. You should upload this as a separate file labeled ‘Manuscript’.

If you would like to make changes to your financial disclosure, please include your updated statement in your cover letter. Guidelines for resubmitting your figure files are available below the reviewer comments at the end of this letter.

If applicable, we recommend that you deposit your laboratory protocols in protocols.io to enhance the reproducibility of your results. Protocols.io assigns your protocol its own identifier (DOI) so that it can be cited independently in the future. For instructions see: https://journals.plos.org/plosone/s/submission-guidelines#loc-laboratory-protocols. Additionally, PLOS One offers an option for publishing peer-reviewed Lab Protocol articles, which describe protocols hosted on protocols.io. Read more information on sharing protocols at https://plos.org/protocols?utm_medium=editorial-email&utm_source=authorletters&utm_campaign=protocols.

As the corresponding author, your ORCID iD is verified in the submission system and will appear in the published article. PLOS supports the use of ORCID, and we encourage all coauthors to register for an ORCID iD and use it as well. Please encourage your coauthors to verify their ORCID iD within the submission system before final acceptance, as unverified ORCID iDs will not appear in the published article. *Only*  the individual author can complete the verification step; PLOS staff *cannot*  verify ORCID iDs on behalf of authors.

We look forward to receiving your revised manuscript.

Kind regards,

Omid Beiki, MD, PhD, MSc

Academic Editor

PLOS One

Journal Requirements:

1. If the reviewer comments include a recommendation to cite specific previously published works, please review and evaluate these publications to determine whether they are relevant and should be cited. There is no requirement to cite these works unless the editor has indicated otherwise.

2. Please review your reference list to ensure that it is complete and correct. If you have cited papers that have been retracted, please include the rationale for doing so in the manuscript text, or remove these references and replace them with relevant current references. Any changes to the reference list should be mentioned in the rebuttal letter that accompanies your revised manuscript. If you need to cite a retracted article, indicate the article’s retracted status in the References list and also include a citation and full reference for the retraction notice.

[Note: HTML markup is below. Please do not edit.]

Reviewers’ comments:

Reviewer’s Responses to Questions

**Comments to the Author**

1. Does the manuscript provide a valid rationale for the proposed study, with clearly identified and justified research questions?

Reviewer #1: Partly

Reviewer #2: Yes

**********

2. Is the protocol technically sound and planned in a manner that will lead to a meaningful outcome and allow testing the stated hypotheses?

Reviewer #1: Partly

Reviewer #2: Partly

**********

3. Is the methodology feasible and described in sufficient detail to allow the work to be replicable?

Reviewer #1: Yes

Reviewer #2: Yes

**********

4. Have the authors described where all data underlying the findings will be made available when the study is complete?

The PLOS Data policy

Reviewer #1: Yes

Reviewer #2: No

**********

5. Is the manuscript presented in an intelligible fashion and written in standard English?

Reviewer #1: Yes

Reviewer #2: Yes

**********

Please use the space provided to explain your answers to the questions above and, if applicable, provide comments about issues authors must address before this protocol can be accepted for publication. You may also include additional comments for the author, including concerns about research or publication ethics.

You may also provide optional suggestions and comments to authors that they might find helpful in planning their study.

Reviewer #1: The authors have addressed most of the comments raised. However, there are few comments from Reviewer 2 that have not been addressed. The authors are encouraged to respond to all outstanding comments.

Reviewer #2: Interesting study planned here. The revised manuscript is improved but a few minor comments from these revisions:

1. The new progression table need numbering. I was also concerned about the Red for drop out being 3+ by 9 months, as that’s quite common drop out rate for feasibility studies so does that mean you would not continue if only 3 people dropped out by 9 months (also is that per arm or overall?). It may be better to try to review/design mitigation for main if only a few dropping out, or otherwise provide justification in footnotes of table for the rationale for this criteria

2. I’m still unconvinced MICE would be helpful here- would it be for the proposed RCT outcome assessment only? At the target sample size of 32, a MICE procedure- particularly one fitted separately by treatment arm- is likely to be unstable especially involving categorical variables. if included maybe the authors could provide some further details on the variables and conditional models etc, and presented as auxiliary. I think primary missingness analysis should be description of the amount, patterns and reasons for missing and evaluation of whether any candidate variables can be collected for future trials.

3. You may want to add plans for making the data from the feasibility study available.

**********

what does this mean?). If published, this will include your full peer review and any attached files.

If you choose “no”, your identity will remain anonymous but your review may still be made public.

**Do you want your identity to be public for this peer review?**  For information about this choice, including consent withdrawal, please see our Privacy Policy

Reviewer #1: No

Reviewer #2: **Yes:** Jenny Harris

**********

[NOTE: If reviewer comments were submitted as an attachment file, they will be attached to this email and accessible via the submission site. Please log into your account, locate the manuscript record, and check for the action link "View Attachments". If this link does not appear, there are no attachment files.]

To ensure your figures meet our technical requirements, please review our figure guidelines: https://journals.plos.org/plosone/s/figures

You may also use PLOS’s free figure tool, NAAS, to help you prepare publication quality figures: https://journals.plos.org/plosone/s/figures#loc-tools-for-figure-preparation.

NAAS will assess whether your figures meet our technical requirements by comparing each figure against our figure specifications.

# Author comment: Author response to Decision Letter 2

**DOI:** 10.1371/journal.pone.0350956.r004

24 Aug 2026

Table 1: Response to second review

Reviewer 1: Not applicable

Reviewer 2

The new progression table needs number Table numbering has been added. The table is now cited in the text. Outcomes, page 12.

Concern that progression “Red” for 3+ participant drop out by 3 months. We have updated the “Red” criteria to be “not applicable” with a footnote explaining that if drop out exceeds 2, we will undertake design review. Outcomes pages 12-13

Sample size is too small for MICE analysis of missing data. We have changed the missing data analysis to be descriptive and informative of future trials. Procedure, pages 17-18

Make plans for making data from the feasibility study available We have indicated that feasibility assessment data will be available on appropriate request. Title page, page 2

Attachment Submitted filename: FLoC Manuscript Response to Reviewers Table_R2.docx

# Editor report: Decision Letter 2

**DOI:** 10.1371/journal.pone.0350956.r005

**Authors:** Omid Beiki

26 Aug 2026

An Adapted Enhanced Social Prescribing Intervention vs. Usual Care in the Treatment of Loneliness in Cancer Survivors: Study Protocol for a Feasibility Pilot Randomized Controlled Trial

PONE-D-26-13807R2

Dear Dr. Cole,

We’re pleased to inform you that your manuscript has been judged scientifically suitable for publication and will be formally accepted for publication once it meets all outstanding technical requirements.

Within one week, you’ll receive an e-mail detailing the required amendments. When these have been addressed, you’ll receive a formal acceptance letter and your manuscript will be scheduled for publication.

An invoice will be generated when your article is formally accepted. Please note, if your institution has a publishing partnership with PLOS and your article meets the relevant criteria, all or part of your publication costs will be covered. Please make sure your user information is up-to-date by logging into Editorial Manager at Editorial Manager® and clicking the ‘Update My Information’ link at the top of the page. For questions related to billing, please contact billing support.

If your institution or institutions have a press office, please notify them about your upcoming paper to help maximize its impact. If they’ll be preparing press materials, please inform our press team as soon as possible -- no later than 48 hours after receiving the formal acceptance. Your manuscript will remain under strict press embargo until 2 pm Eastern Time on the date of publication. For more information, please contact onepress@plos.org.

Kind regards,

Omid Beiki, MD, PhD, MSc

Academic Editor

PLOS One

Additional Editor Comments (optional):

Reviewers’ comments:

# Editor report: Acceptance letter

**DOI:** 10.1371/journal.pone.0350956.r006

**Authors:** Omid Beiki

PONE-D-26-13807R2

PLOS One

Dear Dr. Cole,

I’m pleased to inform you that your manuscript has been deemed suitable for publication in PLOS One. Congratulations! Your manuscript is now being handed over to our production team.

At this stage, our production department will prepare your paper for publication. This includes ensuring the following:

* All references, tables, and figures are properly cited

* All relevant supporting information is included in the manuscript submission,

* There are no issues that prevent the paper from being properly typeset

You will receive further instructions from the production team, including instructions on how to review your proof when it is ready. Please keep in mind that we are working through a large volume of accepted articles, so please give us a few days to review your paper and let you know the next and final steps.

Lastly, if your institution or institutions have a press office, please let them know about your upcoming paper now to help maximize its impact. If they’ll be preparing press materials, please inform our press team within the next 48 hours. Your manuscript will remain under strict press embargo until 2 pm Eastern Time on the date of publication. For more information, please contact onepress@plos.org.

You will receive an invoice from PLOS for your publication fee after your manuscript has reached the completed accept phase. If you receive an email requesting payment before acceptance or for any other service, this may be a phishing scheme. Learn how to identify phishing emails and protect your accounts at https://explore.plos.org/phishing.

If we can help with anything else, please email us at customercare@plos.org.

Thank you for submitting your work to PLOS One and supporting open access.

Kind regards,

PLOS One Editorial Office Staff

on behalf of

Dr. Omid Beiki

Academic Editor

PLOS One
